# Evidence for microbially-mediated tradeoffs between growth and defense throughout coral evolution

**DOI:** 10.1186/s42523-024-00370-z

**Published:** 2025-01-03

**Authors:** Hannah E. Epstein, Tanya Brown, Ayọmikun O. Akinrinade, Ryan McMinds, F. Joseph Pollock, Dylan Sonett, Styles Smith, David G. Bourne, Carolina S. Carpenter, Rob Knight, Bette L. Willis, Mónica Medina, Joleah B. Lamb, Rebecca Vega Thurber, Jesse R. Zaneveld

**Affiliations:** 1 School of Life Sciences, University of Essex, Wivenhoe Park, Colchester, Essex CO4 3SQ UK; 2 Department of Microbiology, Oregon State University, 226 Nash Hall, Corvallis, OR 97331 USA; 3 School of Science, Technology, Engineering, and Mathematics, Division of Biological Sciences, University of Washington Bothell, UWBB-277, Bothell, WA 98011 USA; 4 Department of Biology, University of Texas, Tyler, TX 75799 USA; 5 Department of Ecology and Evolutionary Biology, University of California, Irvine, CA 92697 USA; 6 Center for Global Health and Infectious Diseases Research, University of South Florida, 13201 Bruce B. Downs Blvd, MDC 56, Tampa, FL 33612 USA; 7 Department of Biology, Pennsylvania State University, 208 Mueller Lab, University Park, Philadelphia, PA 16802 USA; 8 Hawaiʻi & Palmyra Program, The Nature Conservancy, Honolulu, HI USA; 9 School of Pharmacy, University of Washington, Seattle, WA 98195 USA; 10 College of Science and Engineering, James Cook University, Townsville, QLD 4811 Australia; 11 Australian Institute of Marine Science, Townsville, QLD 4810 Australia; 12 Scripps Institution of Oceanography, University of California, San Diego, La Jolla, CA 92093 USA; 13 Center for Microbiome Innovation, University of California, San Diego, La Jolla, CA 92093 USA; 14 Department of Pediatrics, University of California, San Diego, La Jolla, CA 92093 USA; 15 Department of Computer Science & Engineering, University of California, San Diego, La Jolla, CA 92093 USA; 16 Shu Chien-Gene Lay Department of Bioengineering, University of California, San Diego, La Jolla, CA 92093 USA; 17 Halıcıoğlu Data Science Institute, University of California, San Diego, La Jolla, CA 92093 USA; 18 ARC Centre of Excellence for Coral Reef Studies, James Cook University, Townsville, QLD 4811 Australia

**Keywords:** Evolutionary tradeoffs, Evolution, Coral disease, Coral microbiome, Coral reefs

## Abstract

**Background:**

Evolutionary tradeoffs between life-history strategies are important in animal evolution. Because microbes can influence multiple aspects of host physiology, including growth rate and susceptibility to disease or stress, changes in animal-microbial symbioses have the potential to mediate life-history tradeoffs. Scleractinian corals provide a biodiverse, data-rich, and ecologically-relevant host system to explore this idea.

**Results:**

Using a comparative approach, we tested if coral microbiomes correlate with disease susceptibility across 425 million years of coral evolution by conducting a cross-species coral microbiome survey (the “Global Coral Microbiome Project”) and combining the results with long-term global disease prevalence and coral trait data. Interpreting these data in their phylogenetic context, we show that microbial dominance predicts disease susceptibility, and traced this dominance-disease association to a single putatively beneficial symbiont genus, *Endozoicomonas. Endozoicomonas* relative abundance in coral tissue explained 30% of variation in disease susceptibility and 60% of variation in microbiome dominance across 40 coral genera, while also correlating strongly with high growth rates.

**Conclusions:**

These results demonstrate that the evolution of *Endozoicomonas* symbiosis in corals correlates with both disease prevalence and growth rate, and suggest a mediating role. Exploration of the mechanistic basis for these findings will be important for our understanding of how microbial symbioses influence animal life-history tradeoffs.

**Supplementary Information:**

The online version contains supplementary material available at 10.1186/s42523-024-00370-z.

## Introduction

Tradeoffs in life-history strategy are key features in animal evolution [1, 2]. These tradeoffs often involve differential investments in life-history traits such as growth rate [3]; reproductive maturation, timing, and fecundity [4]; or resistance to stress [5], predation [6], or disease [7]. The fitness costs and benefits of these investments are often context-dependent and shifts in ecological or environmental conditions can favor some life-history strategies over others [5], sculpting trait evolution within animal lineages and reshaping ecological communities. Global climate change is shifting the patterns and prevalence of disease in many animal taxa, while increasing the virulence of some pathogens [8, 9]. Identifying evolutionary tradeoffs and resulting trait correlations associated with disease susceptibility [10] can therefore help predict how species survival will shift with climate change.

Although much research on evolutionary tradeoffs focuses on the traits of animals themselves, it is also well documented that the physiology [11], fitness and even behavior [12] of many animals are influenced by their microbiomes. Animal microbiomes have been linked to multiple key life-history traits, including growth [13], development rate [13], fecundity [13], stress resistance [11, 14], and disease susceptibility [14]. It therefore seems likely that microbial symbiosis is an important aspect of animal life-history tradeoffs and may correlate with host traits over long periods of animal evolution. However, testing the potential relevance of microbial symbiosis for life-history strategy evolution over long time periods is challenging.

The reef-building corals that have evolved over 425 million years represent a diverse group of animals, including an estimated >1600 species [15], with an extensive fossil record, and a well-known variety in both life-history strategy [2] and microbial symbiosis [16–18]. As such, they present a valuable opportunity to explore connections between microbes and life history strategy. These animals also have special ecological and societal importance, as corals are foundational to reef ecosystems that support some of the most biodiverse assemblages on the planet and the livelihoods of many coastal communities [19]. Yet the ancient diversity of coral reefs is currently threatened by global climate change, which is driving both dramatic mass bleaching events and increased prevalence and severity of disease outbreaks [8].

Alongside research on how coral health is affected by both well-studied (e.g., Symbiodiniaceae [20–22]) and emerging (e.g., corallicolids, fungi [23]) microbial eukaryotes, extensive research has demonstrated that present-day communities of coral-associated bacteria and archaea (hereafter ‘coral microbiomes’) play a myriad of roles in host biology that could impact disease susceptibility. These include antimicrobial production [24], predation of pathogens [25], jamming of quorum-sensing systems [26], and passive competition for space and resources. Yet these microbiomes are also influenced by host traits [16], local environmental factors, and ecological context [27], including host disease susceptibility patterns within and among species [28]. While this supports a connection between present-day coral life-history, microbiome structure and disease susceptibility, these data do not directly allow for statistical testing of evolutionary hypotheses about potential roles of microbial symbiosis in life history tradeoffs.

Clarifying whether microbiome structure and coral life-history traits correlate over coral evolution globally will contextualize studies of extant coral symbiosis and disease at local or regional scales. Several lines of research have created a strong foundation on which such comprehensive comparative evolutionary analyses can be built. Coral disease patterns have been intensively researched, and an increasing number of datasets are now openly available [29]. Well-curated global databases of coral physiological traits [30] have been established and mapped to coral life-history strategies [2]. Finally, several large cross-species studies of corals and their microbiomes have been launched. These advances provide an opportunity to compare host trait data and microbiome structure from across the coral tree of life.

**Fig. 1:**
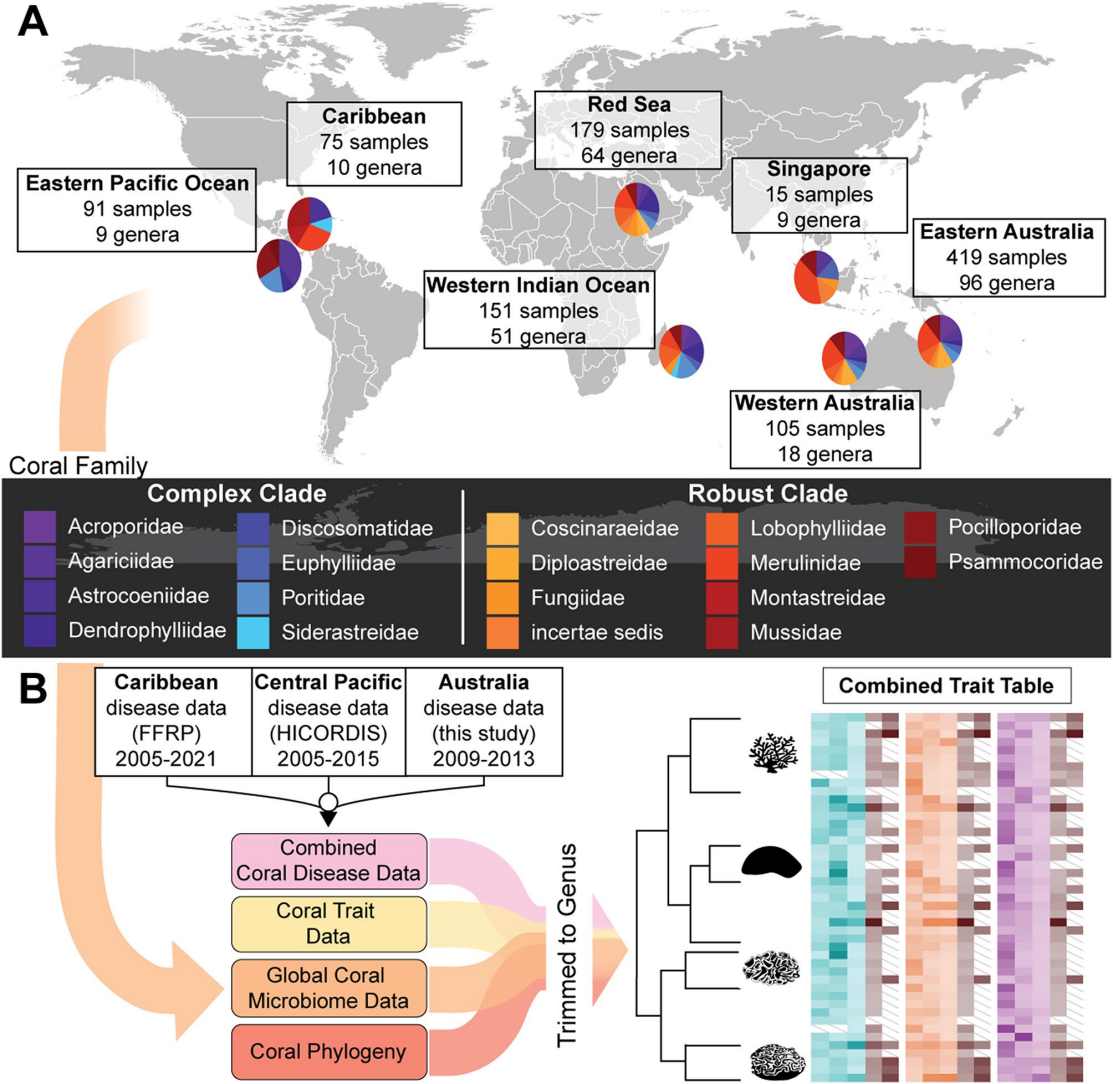
Conceptual overview of data sources integrated for the project. (**A**) Map of sampling locations for coral microbiomes analyzed in the manuscript. Pie charts show the proportion of coral samples from families in the Complex clade (cool colors) and Robust clade (warm colors). Samples were collected from coral mucus, tissue, and endolithic skeleton (see Methods). (**B**) Schematic representation of data integration for the project. Coral microbiome data (as shown in A) were combined with long-term disease prevalence data from 3 projects (the Florida Reef Resilience Program (FFRP), the Hawaiʻi Coral Disease Database (HICORDIS), and data from Australia (this study)), as well as coral trait data from the Coral Trait Database, and a molecular phylogeny of corals (see Methods). To integrate data from these disparate sources, all annotations were pooled at the genus level. The end product was a trait table of microbiome, taxonomic, physiological, and disease data across diverse coral genera

Here, we test whether microbiome structure correlates with two key aspects of coral life history strategy: disease susceptibility and growth rate. To address this question quantitatively, we first characterized the microbiome composition from visibly healthy samples of 40 coral genera using 16S rRNA gene amplicon sequencing results from the Global Coral Microbiome Project [16] (Supplementary Data Table S1a), and subsequently combined these data with coral growth rates from the Coral Trait Database [30], and genus-level long-term disease prevalence data from several tropical regions around the globe (Fig. 1). These long-term disease datasets included the Florida Reef Resilience Project data (FRRP, https://frrp.org/*)*, Hawaiʻi Coral Disease Database (HICORDIS) [29], and new data covering eastern Australia (this study; Supplementary Data Table S1b). With the resulting microbiome structure, coral growth rate, and disease data across a global distribution of coral genera (Supplementary Data Table S1c), we compared these traits using methods that account for phylogenetic correlations using a time-calibrated multi-gene reference tree of corals [31].

## Results

### Coral microbiomes are dominated by a small number of bacterial taxa

**Fig. 2:**
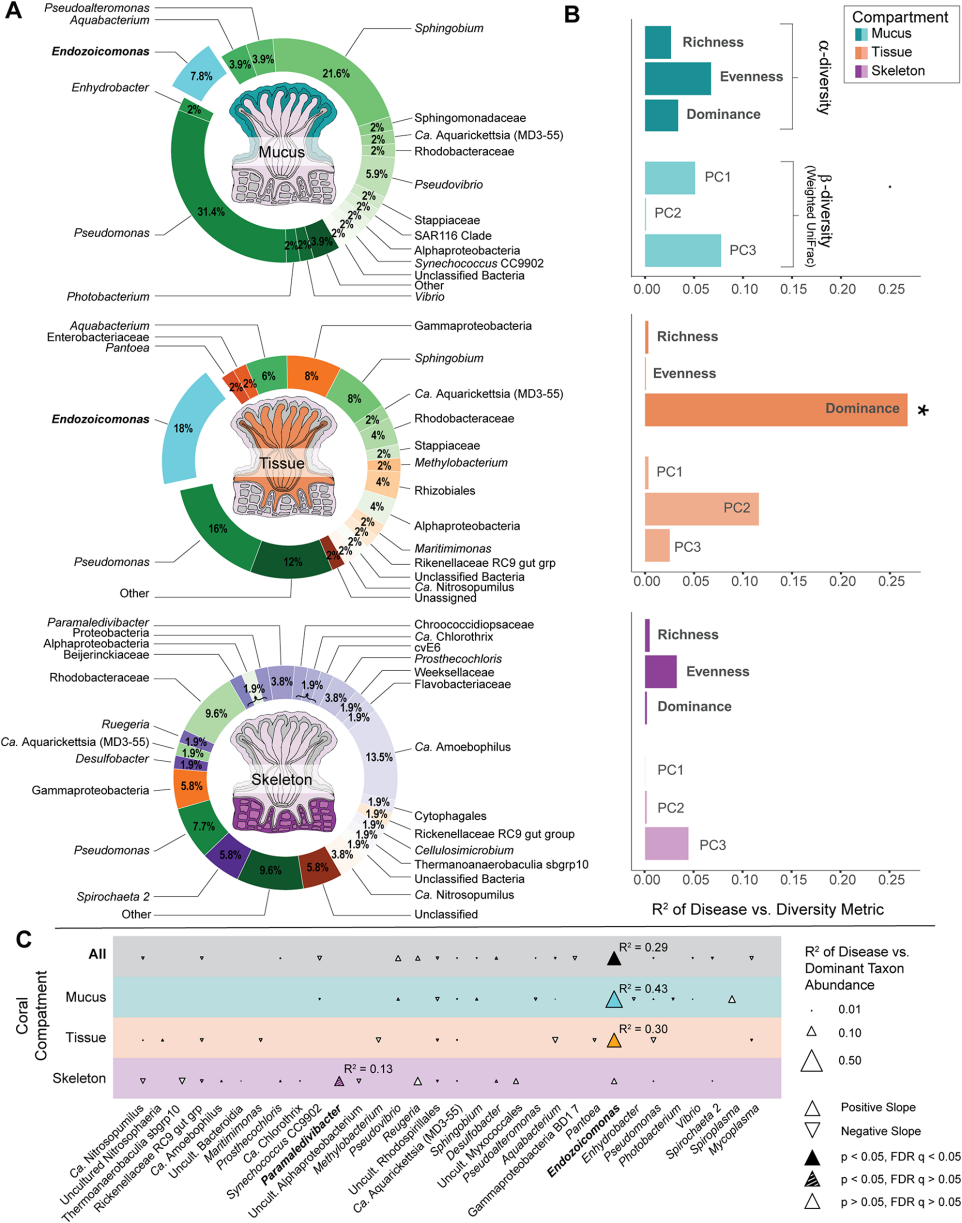
Dominant microbes in the coral microbiome. (**A**) Dominant bacterial or archaeal genera in coral mucus (cyan), tissue (orange), or skeleton (purple) microbiomes. Pie wedges represent the fraction of coral host genera in which the labeled bacterium is more abundant than all other bacterial or archaeal taxa. Cyan shades represent microbes dominant in mucus, oranges represent microbes dominant in tissue (but not mucus), purple shades represent microbes dominant in skeleton (but not mucus or tissue). *Endozoicomonas*, which is of special significance later in the paper, is highlighted in aqua. (**B**) Bar charts showing correlations between microbiome alpha and beta diversity metrics and disease, represented by the R^2^ for PGLS correlations. Alpha diversity metrics include richness, evenness (Gini index), and dominance (Simpson’s index), and weighted UniFrac beta diversity metrics including the three principal component axes (PC1, PC2, PC3) that represent measures of community structure. Significant relationships (*p* < 0.05, Supplementary Data Table S4) are marked by an asterisk (*). (**C**) Bubble plot showing correlations between dominant microbial taxa and coral disease prevalence. The size of each triangle represents the R^2^ for PGLS correlations between disease susceptibility and microbial relative abundance for each listed taxon in either all samples (top row), mucus samples (cyan row), tissue samples (orange row), or skeleton samples (purple row). Colored points were significant (*p* < 0.05, FDR q < 0.05) and hashed points were nominally significant (*p* < 0.05, FDR q > 0.05; Supplementary Data Table S7a). Points that were not significant or had too little data (*n* < 5) for reliable testing are marked in white. Taxa whose relative abundance is significantly correlated with disease are marked in bold on the x-axis

The microbiome of corals is often dominated by a few highly-abundant taxa that demonstrate species-specificity [17, 18], though why these highly-abundant microbial taxa differ across coral diversity is unknown. To test this, we first identified a restricted set of dominant bacterial or archaeal taxa in visibly healthy corals retrieved from mucus, tissue, and skeleton samples of 40 coral genera. (‘Dominant taxa’ were defined as those that are most abundant on average within all samples from a given portion of coral anatomy in a given coral genus). Thirty-eight of the coral genera were dominated by the bacterial classes 𝛼 - or γ-proteobacteria, which are known to include common coral associates [17], with more detailed taxonomy revealing that the number of dominant bacterial and archaeal genera across compartments is also somewhat limited (Fig. 2A; Supplementary Data Table S2). For example, only 17 genera of bacteria or archaea accounted for the dominant microbial genus in the tissue microbiomes of all 40 coral genera (this number excludes 4 unclassified ‘genera’ that could not be classified to at least the order level). Mucus and skeleton showed similar trends, with only 16 and 25 dominant genera, plus 2 or 4 unclassified genera, respectively. Across coral-associated bacterial or archaeal genera, *Pseudomonas* was most commonly dominant in mucus (31.4% of coral genera), while *Endozoicomonas* was most commonly dominant in tissue (18%) and *Candidatus* Amoebophilus (13.5%) was most commonly dominant in skeleton microbiomes. However, whether differences in microbiome structure and dominant microbes across coral diversity influence differences in coral physiology is not yet well understood.

### Microbiome dominance correlates with coral disease susceptibility

We visualized the evolution of coral disease susceptibility and multiple measures of microbiome diversity using ancestral state reconstruction (Supplementary Figs. S1 & S2), then tested whether microbial alpha or beta diversity correlated with disease susceptibility using phylogenetic generalized least squares (PGLS). We found no evidence for an effect of microbiome ecological richness or evenness (considered individually) on disease susceptibility (Supplementary Data Table S3), and limited evidence for an effect of microbiome composition on disease susceptibility (Supplementary Information; Supplementary Data Table S4). However, given that cross-species differences in a limited number of dominant microbes were very notable in the data, we hypothesized that corals with highly abundant bacterial taxa might display more disease vulnerability. To quantify this, ecological dominance among identified amplicon sequence variants (ASVs) was calculated using Simpson’s Index, which estimates the probability that two species drawn from a population belong to the same group, and thereby incorporates aspects of both richness and evenness simultaneously. We correlated Simpson’s Index against coral disease prevalence for either all coral samples, or those in mucus, tissue, or skeleton considered individually. In coral tissue, microbiome dominance significantly correlated with disease, explaining roughly 27% of overall variation in disease susceptibility across coral species (PGLS: R^2^ = 0.27, *p* = 0.0006, FDR q = 0.025; Supplementary Data Table S3a; Supplementary Fig S1C). No other combination of alpha diversity measure and compartment correlated with disease after accounting for multiple comparisons (Fig. 2B). Thus, microbiome dominance as measured by Simpson’s Index was a far stronger predictor of coral disease susceptibility than 𝛼 -diversity measures that considered either richness or evenness individually. Regionally-specific analysis, which eliminates potential confounders due to the global nature of the comparison, recaptured this dominance-disease relationship (Supplementary Information; Supplementary Data Table S3b). Further testing showed that corals dominated by γ-proteobacteria drove the dominance-disease trend, suggesting a specific microbial genus (rather than a general ecological feature) might be responsible for this striking correlation (Supplementary Information; Supplementary Table S3c).

### The coral symbiont *Endozoicomonas* drives dominance-disease correlations

**Fig. 3:**
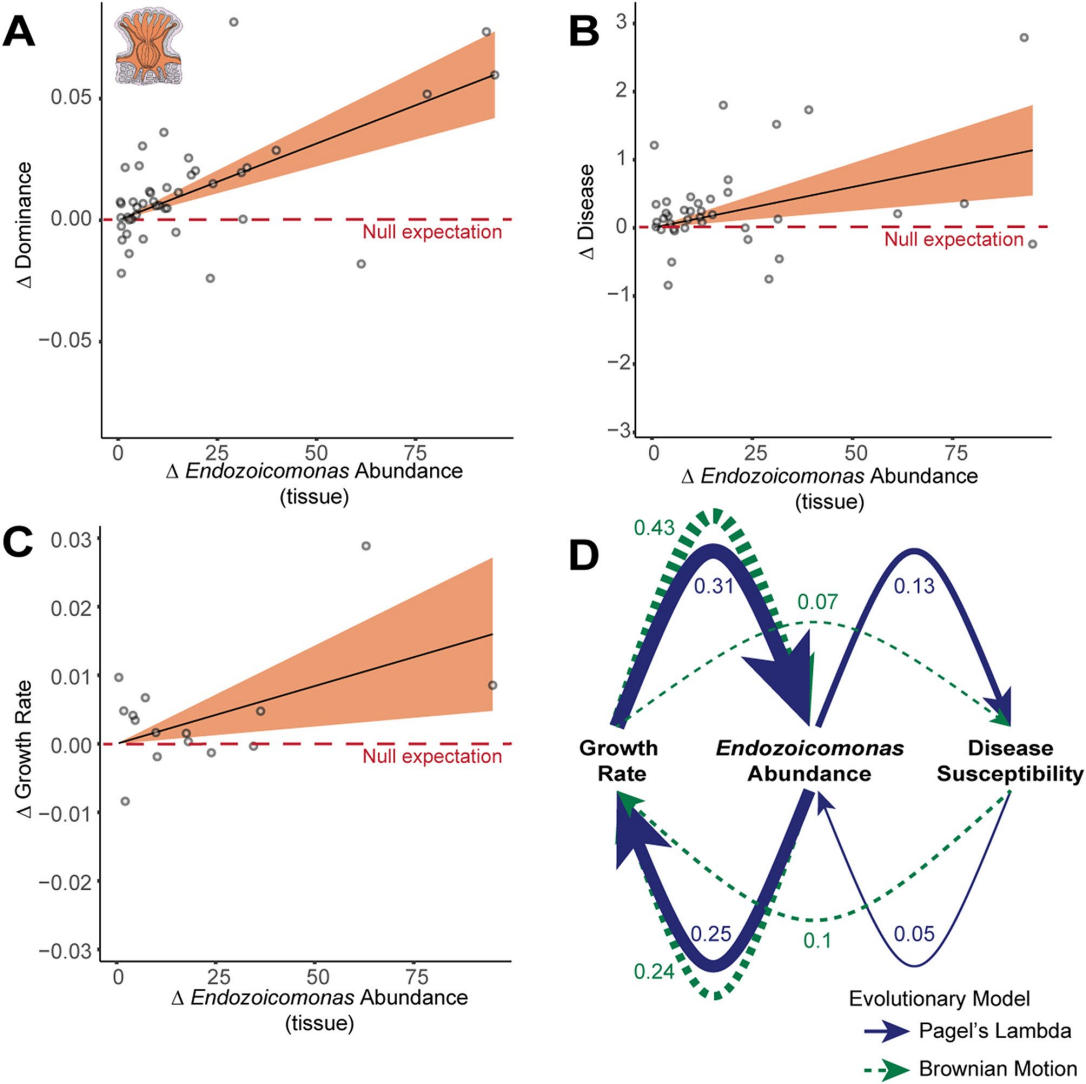
*Endozoicomonas* correlates with growth and disease. Phylogenetic independent contrast in *Endozoicomonas* relative abundance in coral tissue (per 1000 reads), correlated against (**A**) contrast in microbial dominance in coral tissue (assessed by Simpson’s Index), (**B**) constrast in coral disease susceptibility (estimated from integrated long-term coral disease prevalence data) and (**C**) coral growth rate (mm per year) from the Coral Traits Database. Dotted red lines in panels A-C indicate the null expectation that if traits are uncorrelated, change in the x-axis trait will not correlate with changes in the y-axis trait, with contrasts instead distributed equally above or below the dotted line. Statistics from phylogenetic generalized least squares (PGLS) regression for A-C are available in Supplemental Data Tables 5 and 9. (**D**) Modeled strength and direction of causality between *Endozoicomonas* relative abundance, disease susceptibility and growth rate during coral evolution using both Brownian Motion (blue) and Pagel’s Lambda (green, dotted) evolutionary models. The thickness of the lines represents the averaged standardized path coefficients of the top competing models based on CICc values (Supplementary Data Table S11)

Bacteria in the genus *Endozoicomonas* are among the most-studied γ-proteobacterial symbionts of corals. In several species *Endozoicomonas* forms prominent aggregates known as CAMAs (coral associated microbial aggregates) in coral tissue [32]. In species where members of genus *Endozoicomonas* are common, decreases in relative abundance during coral bleaching or disease are frequently observed [33], suggesting a commensal or mutualistic rather than opportunistic relationship with host health, although evidence exists for the potential of *Endozoicomonas* to form relationships with corals along the entire spectrum of symbioses (i.e., beneficial, commensal, and/or antagonistic; see [34]). Further, it has previously been observed that the family Endozoicomonadaceae shows by far the strongest signal of cophylogeny with coral hosts among tested bacterial families in coral tissue [16]. In the present dataset, *Endozoicomonas* was also the single genus that most typically dominated coral tissue microbiomes (18% of coral genera; Fig. 2A). We therefore tested whether the signal of microbiome dominance on disease susceptibility could be explained by the abundances of dominant taxa, and found that across all corals in our dataset (regardless of whether *Endozoicomonas* was present and/or dominant; *n* = 40 genera), *Endozoicomonas* relative abundance explained the majority of variation in ecological dominance among coral tissue microbiomes (PGLS: R^2^: 0.60, *p* = 6.2 × 10^− 10^, FDR q = 2.5 × 10^− 9^; Figs. 2C and 3A; Supplementary Data Table S5a). Further, the relative abundance of *Endozoicomonas* in coral tissue alone explained 30% of variance in overall disease susceptibility (PGLS: R^2^ = 0.30, *p* = 0.0002, FDR q = 0.0004; Fig. 3B; Supplementary Data Table S5a), exceeding the signal from ecological dominance. *Endozoicomonas* remained significantly correlated with disease susceptibility after testing multiple linear models with depth, temperature, extent of turf algae contact, latitude and overall microbiome richness as confounders (Supplementary Data Table S5b & c). Neither commonly opportunistic microbes in corals (Supplementary Data Table S6), nor other dominant microbes (Supplementary Data Table S7) showed similar patterns (Supplementary Information). Thus, our prior results linking ecological dominance and overall disease susceptibility appear to be largely explained by changes in *Endozoicomonas* relative abundance over coral evolution.

### *Endozoicomonas* is associated with high growth rates

*Endozoicomonas* is often linked to metabolic benefits to the coral host [35, 36] (but see [34]), including a potential role in steroid processing [37]. Experimental studies have shown that decreases in its relative abundance are typical with disease [38, 39] or other health stressors such as bleaching [33]. This suggests that the striking correlation between *Endozoicomonas* and disease is not due to pathogenesis by *Endozoicomonas*. There are several possibilities for how a non-pathogen might nonetheless increase disease, including opportunity costs in host biology (e.g., in innate immunity, permissiveness to CAMA formation), tradeoffs in microbial symbiosis (e.g., dominance of *Endozoicomonas* vs. more diverse and potentially flexible microbiome associates with benefits for pathogen defense or resilience to environmental change), or tradeoffs driven by host physiological changes induced by *Endozoicomonas* (e.g., in steroid hormone processing). However, regardless of mechanism, if maintenance of high relative abundances of *Endozoicomonas* has fitness costs, they may be balanced by benefits to the host – at least under some conditions.

If symbiosis with *Endozoicomonas* did play a causal role in coral life-history tradeoffs, we hypothesized that we would see a positive correlation between a beneficial coral trait and *Endozoicomonas* that counterbalances the correlation between *Endozoicomonas* and disease. Given that *Endozoicomonas* is thought to be a metabolic mutualist of corals, and it has recently been suggested to facilitate faster coral growth [32], growth rate seemed like a likely candidate for a potential benefit explaining the persistence of coral-*Endozoicomonas* associations. Depending on the mechanism of action, any such *Endozoicomonas* - growth correlations might depend merely on the presence of *Endozoicomonas*, or alternatively on its relative abundance. Using data from the Coral Trait Database (CTDB) [30] we tested whether *Endozoicomonas* relative abundance was correlated with growth rate in corals where we detected *Endozoicomonas* (i.e., the effect of relative abundance alone) and in all corals (i.e., the combined effect of presence and relative abundance). In both cases, we limited this analysis to only corals with replicated growth rate data ( > = 5 replicates in the CTDB).

While the relative abundance of *Endozoicomonas* was not correlated with growth rate across all coral genera (tissue PGLS: R^2^ = 0.11, *p* = 0.17, FDR q = 0.37; Supplementary Data Table S8a), across coral genera where *Endozoicomonas* was detected and replicated growth rate data were available (*n* = 17 genera), its relative abundance in tissue was strongly correlated with growth rate (tissue PGLS: R^2^ = 0.31, *p* = 0.024, FDR q = 0.024; Supplementary Data Table S8b). Unlike for disease susceptibility, several additional microbes showed anatomically-specific correlations with the growth rate of their coral hosts, including strong positive correlations between growth and uncultured *Rhodobacteria* (Family: Terasakiellaceae) and negative correlations between growth rate and the archaeal genus *Nitrosopumilis* (Supplementary Information; Supplementary Data Table S9; Supplementary Fig. S3). However, *Endozoicomonas* appears unique in its association with both growth and disease.

Overall, *Endozoicomonas* may in part explain, or at least correlate with, about a third of known growth rate differences between coral genera. Across the coral genera surveyed in our dataset, initial, low-level symbiosis with *Endozoicomonas* does not correlate with growth rate, but subsequent expansions of the relative abundance of *Endozoicomonas* within coral microbiomes co-occur with both higher average growth rates and greater disease susceptibility.

### *Endozoicomonas* may mediate growth-defense tradeoffs during coral evolution

Having seen that *Endozoicomonas* is correlated with both disease susceptibility and growth-rate in corals, we investigated if these correlations were stronger or weaker than any direct correlation between disease and growth rate in our dataset. Across genera with both growth rate and disease prevalence data, the correlation between growth and disease susceptibility had only a modest effect size and was not statistically significant. Thus, in this dataset *Endozoicomonas* showed stronger associations with both growth and disease than these factors showed with one another, regardless of whether the analysis was conducted across all coral genera (tissue PGLS: R^2^ = 0.12, *p* = 0.17, FDR q = 0.17; Supplementary Data Table S10a) or just those where *Endozoicomonas* was present (tissue PGLS: R^2^ = 0.06, *p* = 0.37, FDR q = 0.37; Supplementary Data Table S10b). This suggested that *Endozoicomonas* relative abundance might not merely mark tradeoffs between growth and disease but may play some causal role in one or both processes.

### Phylogenetic path analysis of growth, disease, and *Endozoicomonas* abundance

The univariate correlations between *Endozoicomonas*, host disease susceptibility and growth rate raise the question of the direction of causality by which these factors have become non-randomly associated during coral evolution. Using phylogenetic path analysis (Methods), we compared 14 models of the relationship between *Endozoicomonas* relative abundance, disease susceptibility, and growth rate (Supplementary Data Table S11a, Fig. Supplementary Fig. S4).

As is common in this type of analysis, more than one model was consistent with the data. However, none of the top models using either Brownian Motion (Supplementary Table S11b) or Pagel’s lambda (Supplementary Data Table S11c) suggested that disease influenced growth rate or vice versa without the influence of *Endozoicomonas* (Fig. 3D), and all significant models include *Endozoicomonas*. Thus, while the precise feedback remains to be determined, causality analysis suggests that, in some capacity, *Endozoicomonas* likely mediates growth rate and disease.

### Diversity within *Endozoicomonas*

Our comparative results across coral genera suggest that the total relative abundance of microbes in genus *Endozoicomonas* is linked to shifts in host disease susceptibility and growth rate over coral evolution. However, *Endozoicomonas* is comprised of many strains that may differ in their interactions with coral hosts. For example, *Endozoicomonas* phylotypes in nearby corals may differ in genomic features like capacity for reactive oxygen species scavenging that could have implications for host-microbial symbiosis [36]. Moreover, our cross-compartment analysis showed anatomically-specific differences in associations between *Endozoicomonas* and host traits: *Endozoicomonas* relative abundances were significantly associated with disease susceptibility and growth rate in tissue, but only disease susceptibility in mucus. In past literature and our results, *Endozoicomonas* are most abundant in tissue [40]. Therefore, differences in associations between host traits and mucus- or tissue-associated *Endozoicomonas* may simply reflect somewhat less statistical power in mucus (where *Endozoicomonas* is less abundant) vs. tissue, and in our growth rate analysis (*n* = 17 genera) vs. disease susceptibility analysis (*n* = 40 genera). However, these results also raise the question of whether stable sub-populations of *Endozoicomonas* in mucus vs. tissue have distinct effects on host physiology.

To test for any differences among mucus- vs. tissue-associated *Endozoicomonas*, we characterized the distribution of *Endozoicomonas* ASVs across coral compartments. Our dataset contained 123 *Endozoicomonas* ASVs. Of these, 23 abundant ASVs explained 95% of total *Endozoicomonas* reads, while the remainder were relatively rare. After removing ASVs with < 10 counts, we sorted the remaining *Endozoicomonas* ASVs according to the compartment in which they showed highest abundance. This yielded 15 ASVs that were most prevalent in mucus, 42 in tissue and 3 in skeleton. We then analyzed the relative abundance of these compartment-specific pools separately to see which, if any, would recapture associations between genus *Endozoicomonas* and host disease susceptibility. In this more nuanced analysis, the pool of *Endozoicomonas* ASVs associated with tissue showed a strong relationship with disease susceptibility (PGLS R^2^ = 0.31, *p* = 0.0002, FDR q = 0.0005), while ASV pools associated with both mucus and skeleton showed no association with disease (PGLS mucus R^2^ = 0.02, *p* = 0.37, FDR q = 0.56; skeleton R^2^ = 0.008, *p* = 0.57, FDR q = 0.57) (Supplementary Data Table S12a). Thus, associations between *Endozoicomonas* relative abundance in mucus and coral disease susceptibility appear to derive from ASVs that have highest relative abundance in bulk tissue samples, but appear in mucus at lower relative abundance – consistent with evidence from fluorescence imaging showing *Endozoicomonas* can aggregate within multiple coral tissues, including tentacles, mesenteries, and calicodermis [32]. In contrast to the strong association between total *Endozoicomonas* relative abundance in coral tissues and host growth rate, the association between tissue-enriched *Endozoicomonas* ASVs and growth rate was not significant in the top model (PGLS FDR q > 0.05; Supplementary Data Table S12b). This may indicate that ASVs excluded in this analysis are important to the *Endozoicomonas* – growth rate association, perhaps due to contributions from ASVs common in multiple compartments or the summed influence of multiple rare ASVs.

Experimental tests on diverse *Endozoicomonas* strains will be important to track the dynamics of *Endozoicomonas* across coral anatomy, and delineate any direct, strain-specific effects on disease susceptibility or growth rate.

## Discussion

We found positive correlations between the total relative abundance of *Endozoicomonas* in coral tissue and the host traits of growth rate and disease susceptibility. This finding complements and contextualizes ongoing work on the mechanisms underlying the coral-*Endozoicomonas* symbiosis [34] and the potential role of *Endozoicomonas* as a metabolic mutualist [32, 35]. It also echoes findings of correlations between life-history strategy and microbiome structure in other important marine invertebrates, such as that between predator defense and microbial abundance in marine sponges [41].

The mechanism by which corals with high proportions of *Endozoicomonas* become more vulnerable to disease are not yet known, but may shed light on their role in coral symbiosis [34]. Because these results rely on relative abundance, it is not yet clear whether differences in absolute abundances of *Endozoicomonas* also vary. Importantly, anatomically-specific variation in true abundances may complicate relative abundance in bulk tissue – for example, if coral taxa vary greatly in absolute microbial abundances outside of CAMAs (similar to low vs. high microbial abundance sponges [42, 43]), those differences could alter apparent *Endozoicomonas* relative abundance. If the pattern of relative abundance reported here corresponds to absolute *Endozoicomonas* abundances, potential explanations fall into three main categories: ecological, structural, or immunological.

Many coral microbes (but not *Endozoicomonas*) are thought to protect against pathogenic disease by mechanisms such as antibiotic secretion [24], direct predation [25], jamming of quorum signaling [26], and through physically occupying space close to host tissues that may restrict binding sites for opportunists and pathogens. In theory, it is possible that high dominance of *Endozoicomonas* may impact the overall diversity or richness of the coral microbiome, effectively restricting the diversity of potential microbial defenses that may benefit the health of the coral. Similarly, *Endozoicomonas* may interact directly or indirectly with other microbiome members in a way that reduces microbially-derived host defenses. However, that *Endozoicomonas* are frequently observed in discrete CAMAs complicates this possibility, as any effects on microbes outside the local area of these CAMAs would have to rely on indirect consequences of *Endozoicomonas*-coral interactions or secreted factors. Nevertheless, if this hypothesis were correct, the reductions in the abundance or relative abundance of *Endozoicomonas* that are often reported in diseased coral phenotypes (e.g., [33]) would then be adaptive on the part of the host, by allowing proportionally greater growth of other, more protective microbes. This hypothesis could be tested by microbial inoculation experiments that increase *Endozoicomonas* abundances prior to or concurrent with disease exposure, with the prediction that this would increase disease severity (although care must be taken to exclude nutritional benefits from corals directly eating the *Endozoicomonas* confounding the results). More systematic studies of whether high abundances of *Endozoicomonas* are exclusively found in visible CAMAs could also speak to the plausibility of this ecological hypothesis, by clarifying the likely routes for interaction between *Endozoicomonas* and other coral-associated microbes.

In addition to ecological interactions, the *Endozoicomonas* - disease susceptibility correlation may also arise as a result of host traits that are permissive for the formation of microbial aggregates. As the cellular processes involved in establishing mutualism, commensalism and pathogenesis often overlap, the same host-microbe interactions that allow *Endozoicomonas* and some other microbes like *Simkania* [32] to aggregate within coral tissues may also be more permissive towards invasion by pathogens. So far known coral pathogens have not been reported to be present within CAMAs. However, other structural mechanisms are possible. For example, the density, morphology, or diversity of septate junctions — which form epithelial barriers similar to tight junctions in chordates [44] — might, in theory, influence the ability of both *Endozoicomonas* and pathogenic microbes to enter coral tissues. This idea could be tested by examining cellular morphology, sequence similarity, and/or gene expression of septate junctions and their constituent components in coral species in which CAMAs did or did not form.

Finally, it is possible that coral immunological strategies that permit symbiosis with high abundances of *Endozoicomonas* also tend to make corals more vulnerable to pathogens. Coral species vary in immune investment (as measured by immune parameters like melanin abundance, phenoloxidase activity, etc.), and low immune investment has been observed to correlate with disease susceptibility [45]. Some theory predicts that the evolution of more permissive immunological strategies is favored by symbionts that provide metabolic benefits to the host [46]. In corals specifically, immune repertoires in key gene families such as TIR-domain containing genes vary greatly between species, which has been hypothesized to influence microbiome structure [47]. Indeed, in sequenced coral genomes the copy number of some of these, such as IL-1R receptors, appear to correlate with several features of coral microbiomes, including *Endozoicomonas* abundance [48]. Thus, symbiosis with *Endozoicomonas* may promote lower immune investment in corals, which in turn increases disease susceptibility. This hypothesis could be tested by comparing the length of coral-*Endozoicomonas* associations, to see whether longer histories of association lead to low immune investment, or by examining selection on innate immune genes in low vs. high *Endozoicomonas* coral lineages (e.g., by dN/dS ratios).

A related immunological explanation would occur if *Endozoicomonas* itself achieves high relative abundances by suppressing aspects of host immunity. Genomic studies of host-associated *Endozoicomonas* identified variation in the proportion of eukaryote-derived genes and domains as a key feature of strain variation, including some domains thought to suppress immunity-induced apoptosis [49]. *Endozoicomonas* has also recently been suggested to play a role in coral hormone homeostasis [37], which could have multiple physiological effects on coral tissues (even those not in direct contact with CAMAs), including potentially influencing both growth rate and immunity. If representatives of diverse strains could be cultured, experiments adding exogenous *Endozoicomonas* might clarify whether *Endozoicomonas* strains have any direct effects on coral immunity, and if so whether they differ from strain to strain.

## Conclusions

Animals evolved in a microbial world. The resulting interactions between animal hosts and their associated microbes influence organismal fitness, and the history of these interactions across generations may influence eco-evolutionary patterns. Using evolutionary analyses of coral microbiomes, we provide evidence that symbiosis with *Endozoicomonas* may mediate growth vs. disease resistance tradeoffs. While further manipulative studies are necessary to confirm this finding and determine the directionality of the relationship, evidence for this trend across the coral tree of life is compelling.

**Fig. 4:**
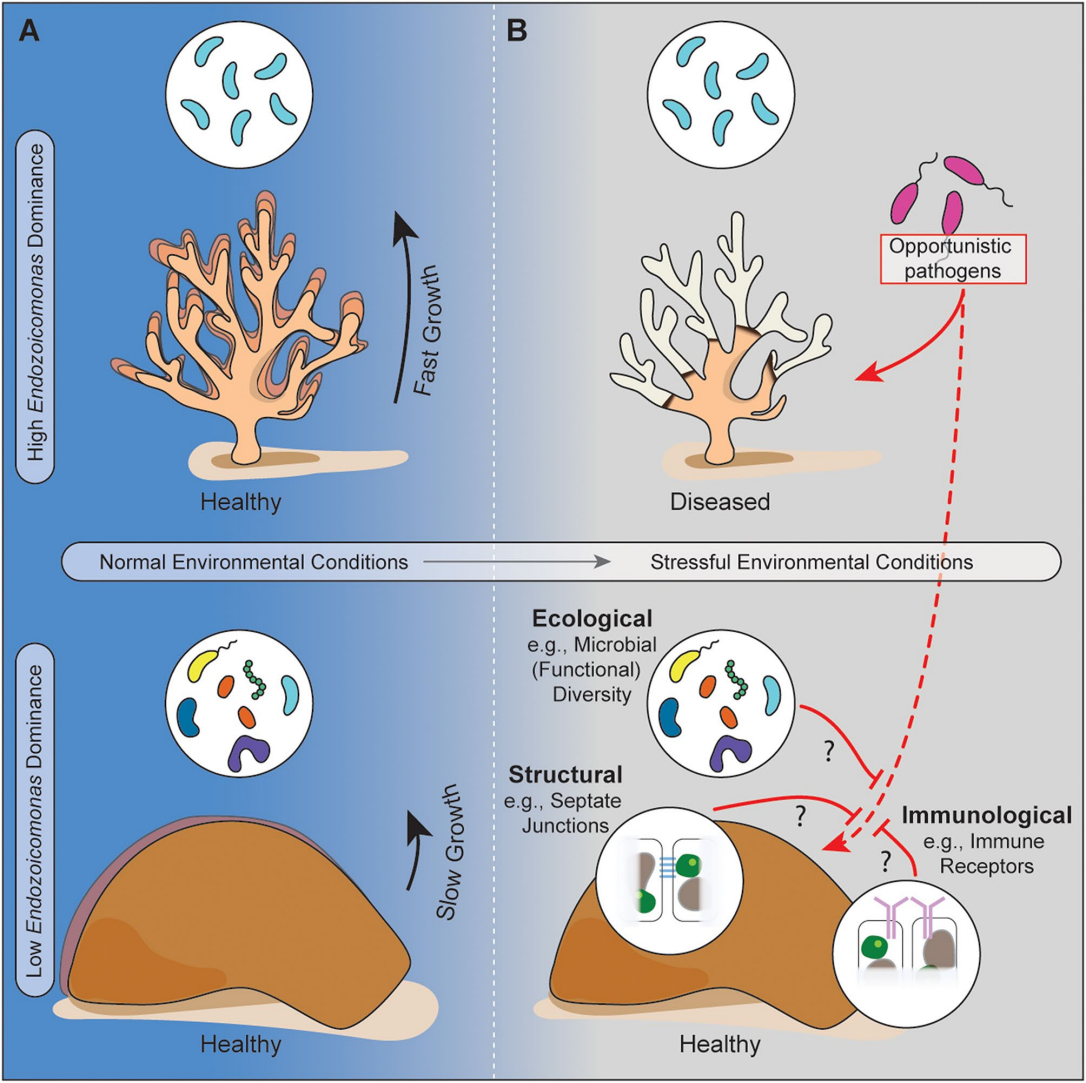
*Endozoicomonas* dominance facilitates life history tradeoffs. Conceptual hypothesis on the role *Endozoicomonas* dominance in coral microbiomes (teal icons, top row) plays in the tradeoff between growth and defense under varying environmental conditions. *Endozoicomonas*-dominated microbiomes may (**A**) provide a metabolic advantage for growth under normal environmental conditions (top left), but (**B**) lack the ecological, structural or immunological defenses against pathogen invasion, and therefore become susceptible to disease under stressful environmental conditions (top right). In contrast, microbiomes not dominated by *Endozoicomonas* (bottom left) grow slower, but may have lower disease susceptibility in stressful environmental conditions (bottom right)

Our comparative approach suggests that *Endozoicomonas*-dominated lineages of corals may grow more quickly under ideal conditions but are more likely to succumb to coral disease. Because much other work has shown that coral disease is exacerbated by global and local stressors such as climate-change driven heat waves or local pollution events [50, 51], this may make *Endozoicomonas-* dominated coral especially vulnerable to environmental change (Fig. 4). It has even been suggested that high dominance of one microbial taxon in the coral microbiome may have a stabilizing effect on the rest of the community [52], thereby limiting the flexibility of the microbiome to functionally adapt through restructuring when exposed to environmental stressors [53].

If microbial symbiosis does play a causal role in coral life history tradeoffs in the present day, then identifying microbes underlying those tradeoffs may benefit microbiome manipulation for targeted coral conservation and restoration strategies. For example, microbial screening (e.g., [54]) could help identify *Endozoicomonas*-dominated coral species or populations that may be more susceptible to disease and drive the conservation and protection of these individuals or their habitats. Identification of these target corals is perhaps most relevant for coral restoration initiatives that include breeding, nursery propagation and out-planting, where coral health is monitored closely and predicting disease susceptibility can inform decision-making. Depending on the mechanism underlying the *Endozoicomonas-*disease susceptibility correlations reported here, *Endozoicomonas*-dominated corals may further represent strong candidates for microbiome engineering (e.g., human-assisted manipulation of host-associated microbes [55] or the application of probiotics [14, 56]) to enhance host resilience in anticipation of stress events by decreasing microbiome dominance. That said, we emphasize that microbiome manipulation and other restoration initiatives are not replacements for efforts to decarbonize global economies to limit greenhouse gas emissions.

The results presented here provide the first evidence of a likely microbe-mediated life-history tradeoff in Scleractinian corals. Further exploration of this and other such potential tradeoffs may shed light on the evolutionary interplay between microbes and the physiology and ecology of their animal hosts.

## Online methods

### Coral sample collection and 16S rRNA pre-processing

16S rRNA sequence data were obtained from visibly healthy coral DNA extractions collected and processed for the Global Coral Microbiome Project (GCMP). This included coral samples taken from Eastern and Western Australia that were used in a previous study by Pollock and co-authors [16] in addition to coral samples taken from the Red Sea, Indian Ocean, Coral Triangle, Caribbean, and Eastern Pacific. All samples compared in this study were collected, processed, and sequenced using consistent protocols as outlined below. In total, 1,440 coral, outgroup, and environmental samples were collected. Of these GCMP samples, the 1,283 scleractinian coral and outgroup samples were used in the present study (Supplementary Data Table S1a). These comprise 132 species and 64 genera of corals originating from 42 reefs spanning the Pacific, Indian, and Atlantic oceans. Excluding outgroups, these data included an average of 22.3 ± 3.3 samples per genus, with a minimum of n of 2 in the genus *Lithophyllon* (Supplementary Data Table S1a, d).

The collection and processing of these coral samples followed the methods outlined in Pollock et al. [16] and are compatible with samples processed for the Earth Microbiome Project [57]. Briefly, three coral compartments were targeted for each sample: tissue, mucus, and skeleton. Mucus was released through agitation of coral surface using a blunt 10mL syringe for approximately 30 s and collected via suction into a cryogenic vial. Small coral fragments were collected by hammer and chisel or bone shears for both tissue and skeleton samples into sterile WhirlPaks (Nasco Sampling, Madison, WI). All samples were frozen in liquid nitrogen on immediate return to the surface prior to processing. In the laboratory, snap frozen coral fragments were washed with sterile seawater and the tissue was separated from skeleton using sterilized pressurized air at between 800 and 2000 PSI. Tissue and skeleton samples were then preserved in PowerSoil DNA Isolation kit (MoBio Laboratories, Carlsbad, CA; now Qiagen, Venlo, Netherlands) bead tubes, which contain a guanidinium preservative, and stored at -80℃ to await further processing. Outgroup non-scleractinian Anthozoans were also opportunistically collected and stored similarly, including healthy samples of the genera *Millepora* (hydrozoan fire coral), *Palythoa* (zoanthid), *Heliopora* (blue coral), *Tubipora* (organ pipe coral), and *Xenia* and *Lobophytum* (soft corals).

Bacterial and archaeal DNA were extracted using the PowerSoil DNA Isolation Kit (MoBio Laboratories, Carlsbad, CA; now Qiagen, Venlo, Netherlands). To select for the 16S rRNA V4 gene region, polymerase chain reaction (PCR) was performed using the following primers with Illumina adapter sequences (underlined) at the 5’ ends: 515 F [58] 5′− TCG TCG GCA GCG TCA GAT GTG TAT AAG AGA CAG GTG YCA GCM GCC GCG GTA A − 3′ and 806R [59] 5’− GTC TCG TGG GCT CGG AGA TGT GTA TAA GAG ACA GGG ACT ACN VGG GTW TCT AAT − 3′). PCR, library preparation, and sequencing on an Illumina HiSeq (2 × 125 bp) was performed by the EMP [57]. All raw sequencing data and associated metadata for the samples used in this study are available on Qiita (qiita.ucsd.edu) under project ID 10895, prep ID 3439.

### Sequence assembly, quality control and taxonomic assignment

16S rRNA sequencing data were processed in Qiita [60] using the standard EMP workflow. Briefly, sequences were demultiplexed based on 12 bp Golay barcodes using “split_libraries” with default parameters in QIIME1.9.1 [61] and trimmed to 100 bp to remove low quality base pairs. Quality control (e.g., denoising, de-replication and chimera filtering) and identification of amplicon sequence variants (ASVs) were performed on forward reads using deblur 1.1.0 [62] with default parameters. The resulting biom and taxonomy tables were obtained from Qiita (CRC32 id: 8817b8b8 and CRC32 id: ac925c85) and processed using a customized QIIME2 v. 2020.8.0 [63] pipeline in python (github.com/zaneveld/GCMP_global_disease). Taxonomic assignment of ASVs was performed using vsearch [64] with SILVA v. 138 [65] (see below).

### Removal of cryptic mitochondrial reads

Coral mitochondrial reads obtained from metaxa2 [66] were added to the SILVA repository to better identify host mitochondrial reads that may be present in the sequencing data [67]. We refer to this expanded taxonomy as “silva_metaxa2” in code. After taxonomic assignment, all mitochondrial and chloroplast reads were removed. The bacterial phylogenetic tree was built using the SATé-enabled phylogenetic placement (SEPP) insertion technique with the q2-fragment-insertion plugin [68] to account for the short-read sequencing data, again using the SILVA v. 138 [65] database as reference taxonomy. The final output from this pipeline consisted of a taxonomy table, ASV feature table and phylogenetic tree that were used for downstream analyses.

### Identification of potential contaminants

Potential contaminants from extraction and sequence blanks (*n* = 103 negative controls) were identified and removed using the decontam package [69] in R v. 4.0.2 [70] with a conservative threshold value of 0.5 to ensure all ASVs that were more prevalent in negative controls than samples were removed (*n* = 662 potential contaminants). The final feature table consisted of a total of 1,383 samples, 195,684 ASVs, and 37,469,008 reads.

### Summary of disease data by coral genus

Disease data were gathered from long-term multi-species surveys in the Florida Keys (the Florida Reef Resilience Program (FRRP), https://frrp.org/), Hawaiʻi (HICORDIS [29]), and Australia (this study). Disease counts for Australian corals were collected over a period of 5 years (2009–2013) across 109 reef sites and 65 coral genera (Supplementary Data Table S1b). At each of the 109 reefs, we surveyed coral health using 3 replicate belt transects laid along reef contours at 3–4 m depth and approximately 20 m apart using globally standardized protocols [71]. Depending on the reef location, belt transects were either 10, 15, or 20 m in length by 2m width making the area surveyed at each reef between 60 and 120m^2^. Within each belt transect, we identified each coral colony over 5 cm in diameter to genus and classified it as either healthy (no observable disease lesions) or affected by one or more of six common Indo-Pacific coral diseases (according to Lamb and co-authors [72]). Together with the FRRP and HICORDIS data, the combined disease dataset contained 582,342 coral observations across 99 coral genera (Supplementary Data Table S1c).

Because many of these disease observations identified corals only to genus, disease prevalence data were summarized at the genus level. All three resources represent coral surveys over time, ranging from 5 to 16 years. We chose such long-term datasets in an attempt to minimize the potential effects of specific events (e.g., bleaching in a single summer) and instead to capture more general trends in disease susceptibility across species, if such trends were present. Summarizing these data at the genus level was thus part of a comparative strategy, enabling us to extract overall trends and average out local circumstances, so that we could find holobiont features that control disease resistance that may protect some corals but not others. When summarizing at the genus level, individual counts of healthy corals or corals with specific diseases were summed within coral genera across these datasets.

To ensure sufficient replication, we excluded coral genera with fewer than 100 observed individuals. This minimal count was selected because it is the lowest frequency at which diseases with a reasonably high frequency (e.g., 5%) can be reliably detected. (With 100 counts, there is a > 95% chance of detecting at least one count of any disease present with > = 5% prevalence; cumulative binomial, 100 trials, success chance = 0.05). Because only very rarely observed taxa were removed, this filtering preserved 99.8% of total observations. Ultimately, our genus-level summary produced a table with 581,311 observations across 60 coral genera (Supplementary Data Table S1d). For a breakdown of disease susceptibility by coral host genus, see Supplementary Fig. S5A.

### Summary of the microbiome data by coral host genus

Statistical summaries of microbiome community composition were calculated for each sample in QIIME2 [63], and then summarized within anatomical compartments and coral genera. These summaries of coral microbiome alpha diversity were richness (observed features per 1000 reads), evenness (the Gini Index), and Simpson’s Index, which combines both richness and evenness. Thus, each combination of coral genus and anatomical compartment — such as *Acropora* mucus — was assigned an average α-diversity value.

Simpson’s Index, which is of particular importance in these results, is at its highest when a single taxon is the only one present in microbiome, and at its lowest when there are both a large number of taxa, and all taxa have equal abundance (or relative abundance). Thus, this measure is reduced both by community richness and community evenness (Simpson’s Index is closely related to Simpson’s Diversity, which is calculated as 1 - Simpson’s Index, such that more rich or even communities produce higher values).

### Construction of a genus level trait table

The summarized, genus-level disease susceptibility data compiled from all disease projects, and the summarized genus-level microbiome diversity data (see above) were combined to form a trait table that was used in subsequent evolutionary modeling. Additionally, the relative abundance of ‘dominant’ microbes analyzed in this study was averaged within genera and added to this genus-level trait table.

### Genus-level summary of a reference coral phylogeny

Starting with a published multigene time-calibrated phylogeny of corals [31] that we had previously used to demonstrate phylosymbiosis in corals [16], we randomly selected one representative species per genus to produce a genus level tree. This approach was preferred over several alternatives — such as trimming the tree back to the last common ancestor of each genus and reconstructing trait values — because it required fewer assumptions about the process of trait evolution. As microbiome data were not available for all genera on the coral tree (e.g., temperate deep-sea corals), the tree was further pruned (preserving branch lengths) to include only the subset of branches that matched those with microbiome data.

### Addition of genus-level coral growth data

To examine the influence of microbiome structure on coral traits, we pulled growth data from the Coral Trait Database [30] from all coral genera that matched those with both microbiome and disease data, and were collected using consistent metrics (mm/yr). This resulted in growth rate data from 18 coral genera that were subsequently combined with our genus-level trait table (Supplementary Data Table S1d; Supplementary Fig. S5B, C).

### Phylogenetic correlative analysis

Shared evolutionary history induces correlations in traits between species that violate the requirement of standard statistical tests that observations must be independent and uncorrelated. Thus, special care must be taken to account for phylogeny in comparative analysis. We first applied Felsenstein’s phylogenetic independent contrasts (PIC) to visualize our cross-genus trait correlations using the phytools R package [73]. This method removes the effect of any shared evolutionary histories by calculating differences in trait values (contrasts) between sister taxa. We next examined the relationships between traits using information-theoretic model selection (that is, comparison of AICc scores) to identify phylogenetic generalized least squares (PGLS) models of evolution that best explained the observed distribution of microbiome α- or β-diversity and disease susceptibility (as continuous evolutionary characters) in extant species. We tested 4 evolutionary models in the caper R package [74]. In the first model, we used PGLS with no branch length transformation (i.e. holding λ, 𝜹, κ = 1). Thus, this first model is equivalent to PIC. In the next 3 models, we transformed branch lengths on the tree by allowing the model to fit either λ, 𝜹, or κ (see below) using maximum likelihood estimation, while fixing the other 2 parameters at 1. We refer to these 4 models as PGLS, PGLS + λ, PGLS + 𝜹, and PGLS + κ. For detailed explanations of each parameter, please refer to Supplementary Data Table S13. Typically, these models estimated very low λ (~ 0), indicating little or low phylogenetic inertia. Multiple comparisons were accounted for by calculating q values for false discovery rate (FDR) control. Significant relationships between the two traits suggests that they are evolutionarily correlated. All statistics reported represent the best PGLS model results.

Additionally, ancestral state reconstructions of key traits were visualized using the contmap function in the phytools R package [73], which in turn estimates internal states using fast maximum-likelihood (ML) ancestral state reconstruct as implemented in the fastAnc phytools function.

Annotated code for all phylogenetic correlations are available within the run_all_PICs.ipynb script on GitHub: https://github.com/zaneveld/GCMP_Global_Disease/blob/master/analysis/core_analysis/procedure/run_all_PICs.ipynb with correlation results and stats organized by analysis number (A1-15).

### Phylogenetic causality analysis

Observing that A and B are correlated famously does not guarantee that A causes B. However, non-random correlation between A and B does imply some causal association - though there are many possibilities (A causes B, B causes A, a positive feedback loop exists between A & B, some external factor C causes both A and B, etc.). Path analysis represents hypotheses of causality using directed acyclic graphs, then tests the different strengths of association predicted under different hypotheses of causation to test which are consistent with data. The cross-species nature of these data further necessitated use of phylogenetic path analysis, which also accounts for expected trait correlations among related genera. Hypotheses of the direction of causality between microbiome (specifically *Endozoicomonas*), disease, and growth rate were tested using a phylogenetic causality analysis performed in the R package phylopath [75]. This analysis tests the ability of different models to explain correlations in trait data. For example, does selection for a high growth rate in turn drive selection for increased *Endozoicomonas* relative abundance, which then increases disease susceptibility, or does symbiosis with *Endozoicomonas* itself separately increase disease and growth? Fourteen potential causality models were tested to incorporate all biologically plausible pathways between *Endozoicomonas* relative abundance, disease susceptibility, and growth rate (Supplementary Data Table S11a; Supplementary Fig. S4). The top performing causality models according to CICc values (using both Pagel’s λ and Brownian Motion models of evolution) were averaged for interpretation and visualization.

### Analysis of *Endozoicomonas* ASVs

ASVs annotated as *Endozoicomonas* at the genus level were extracted from the rarefied QIIME2 coral microbiome feature table. Differences in ASV diversity within the *Endozoicomonas* genus was assessed by PERMANOVA of Weighted UniFrac or Aitchison beta-diversity distance matrices. For analysis of *Endozoicomonas* ASVs by compartment, *Endozoicomonas* ASVs were pooled according to whether they had greatest relative abundance in mucus, tissue or skeleton. The relative abundance of these compartment-specific pools was then regressed against host traits using PGLS, as outlined above.

## Electronic supplementary material

Below is the link to the electronic supplementary material.

Supplementary Material 1

Supplementary Material 2

Supplementary Material 3

Supplementary Material 4

Supplementary Material 5

Supplementary Material 6

Supplementary Material 7

Supplementary Material 8

Supplementary Material 9

Supplementary Material 10

Supplementary Material 11

Supplementary Material 12

Supplementary Material 13

Supplementary Material 14

## Data Availability

All data generated during this study are publicly available and data summaries are included in this publication and its supplementary information files. The raw sequencing data and associated metadata for the coral microbiome samples used in this study are available on Qiita (qiita.ucsd.edu) under project ID 10895, prep ID 3439. Australian disease data (new to this study) are accessible at github.com/zaneveld/GCMP_global_disease and in Supplementary Data Table S1b. Summary data and all associated code for analyses is available at github.com/zaneveld/GCMP_global_disease. Additional publicly available data were used in this study. These include coral disease data from open-access surveys in the Florida Keys (the Florida Reef Resilience Program (FRRP); https://frrp.org/) and Hawaiʻi (HICORDIS; 10.1016/j.dib.2016.07.025), a multigene coral phylogeny published by Huang & Roy 2015 (10.1098/rstb.2014.0010), and coral growth data obtained from the Coral Trait Database (10.1038/sdata.2016.17).
