# Supplementary material for: Evidence for microbially-mediated tradeoffs between growth and defense throughout coral evolution
Source: Anim Microbiome. 2025 Jan 3;7:1. doi: 10.1186/s42523-024-00370-z (PMC11697511; doi:10.1186/s42523-024-00370-z)
Supplement: Supplementary file 1 — Supplementary Material 1 [file 42523_2024_370_MOESM1_ESM.docx]

**Supporting Information for**

**Evidence for microbially-mediated tradeoffs between growth and defense throughout coral evolution**

Hannah E. Epstein^1,2*^, Tanya Brown^3,4^, Ayọmikun O. Akinrinade^3,5^, Ryan McMinds^2,6^, F. Joseph Pollock^7,8^, Dylan Sonett^9^, Styles Smith^7^, David G. Bourne^10,11^, Carolina S. Carpenter^12,13^, Rob Knight^13-16^, Bette L. Willis^10,17^, Mónica Medina^7^, Joleah B. Lamb^5^, Rebecca Vega Thurber^2^, Jesse R. Zaneveld^3^

*^1^School of Life Sciences, University of Essex, Wivenhoe Park, Colchester, Essex CO4 3SQ, UK*

*^2^Department of Microbiology, Oregon State University, 226 Nash Hall, Corvallis, OR 97331, USA*

*^3^School of Science, Technology, Engineering, and Mathematics, Division of Biological Sciences, University of Washington Bothell, UWBB-277, Bothell, WA 98011, USA*

*^4^Department of Biology, University of Texas, Tyler, TX 75799, USA*

*^5^Department of Ecology and Evolutionary Biology, University of California, Irvine, CA 92697, USA*

*^6^Center for Global Health and Infectious Diseases Research, University of South Florida, 13201 Bruce B. Downs Blvd, MDC 56, Tampa, FL 33612, USA*

*^7^Department of Biology, Pennsylvania State University, 208 Mueller Lab, University Park, PA 16802, USA*

*^8^* *Hawaiʻi & Palmyra Program, The Nature Conservancy, Honolulu, HI, USA*

*^9^School of Pharmacy, University of Washington, Seattle, WA 98195, USA*

*^10^College of Science and Engineering, James Cook University, Townsville, Queensland 4811, Australia*

*^11^Australian Institute of Marine Science, Townsville, Queensland 4810, Australia*

*^12^Scripps Institution of Oceanography, University of California, San Diego, La Jolla, CA 92093, USA*

*^13^Center for Microbiome Innovation, University of California, San Diego, La Jolla, CA 92093, USA*

*^14^Department of Pediatrics, University of California, San Diego, La Jolla, CA 92093, USA*

*^15^Department of Computer Science & Engineering, University of California, San Diego, La Jolla, CA 92093, USA*

*^16^Micronoma Inc., San Diego, La Jolla, CA 92121, USA*

*^17^ARC Centre of Excellence for Coral Reef Studies, James Cook University, Townsville, Queensland 4811, Australia*

*Corresponding author: hannah.epstein@essex.ac.uk

**This PDF file includes:**

Supplementary Information

Figures S1 to S4

**Supplementary Information**

**Results**

**Microbiome richness and evenness do not predict disease susceptibility.** To identify how bacterial communities are structured among globally distributed coral taxa, we characterized alpha diversity within the mucus, tissue, and skeleton compartments for each coral genus using several metrics. These included observed features, which measures richness; and the Gini index, which measures evenness. We visualized the evolution of each of these measures of microbiome alpha diversity using ancestral state reconstruction (Figs. S1A, B), then compared them against disease susceptibility using Phylogenetic Generalized Least Squares (PGLS) analysis. While we hypothesized that coral microbiomes high in overall biodiversity might show reduced disease susceptibility — analogous to the ability of more biodiverse ecosystems to resist invasive species^1^ — neither microbiome richness nor evenness were significantly correlated with host disease susceptibility in phylogenetic generalized least squares analysis (PGLS richness vs. disease susceptibility: R^2^ = 0.004, *p* = 0.674, FDR q = 1; PGLS evenness vs. disease susceptibility R^2^ = 0.028, *p* = 0.274, FDR q = 1; Supplementary Data Table 3a). Some specific cases of coral genera with low microbiome richness and high disease susceptibility were identified (i.e., *Pocillopora, Acropora,* and *Montipora*; Figs. S1A, B) but there was no overall trend across all genera surveyed (Fig. 2B). Thus, microbiome richness or evenness alone does not predict coral disease susceptibility.

**The association between microbiome dominance and disease strengthens in regionally-matched data.** The correlation we saw between microbiome dominance and disease persisted in a regionally-matched comparison between disease and microbiome data, and therefore is unlikely to be driven by biogeographic confounders. While the trend between microbiome dominance and coral disease is compelling across our full dataset, not all coral diseases are cosmopolitan and some exist in only one or a few locations^2^. As mismatches between region and disease biogeography could confound our overall results, we sought to assess whether large-scale regional effects drive this trend. For example, perhaps high-dominance corals happen to live in high-disease areas, resulting in incidental correlations between dominance and disease. To test for regional effects, we repeated the PGLS analyses restricting the data to only coral microbiomes from Australia, where sampling was most intensive and for which we have long-term disease datasets best-matched to the microbiome data. In this analysis, ecological dominance in Australian coral tissue microbiomes predicted disease prevalence even more strongly under the lowest AICc model (PGLS: R^2^ = 0.49, p = 0.00015, FDR q = 0.005). However, this correlation was strong under all models (Supplementary Data Table 3b). A likely explanation for this stronger result is simply that the disease and microbiome data were drawn from the same region in this analysis, whereas in other cases the available disease and microbiome data were only partially regionally matched. These stronger results in the Australia-only model suggest that microbiomes vary enough geographically that disease and microbiome data from the same location produce the clearest correlations.

**Microbiome dominance vs. disease correlations are driven by γ-proteobacteria.** Ecological dominance itself seems an unlikely structural property to act as a mechanism of disease resistance. Therefore, we investigated if this high-level summary measure reflected the effects of some specific microbe or set of microbes. For example, disease susceptibility among *Acropora* has been shown to correlate with the abundance of *Rickettsiales* in coral tissues^3,4^.

To test how shifts in the dominant class of microbes in coral tissue interacted with the dominance-disease correlation, we repeated our previous correlations twice: once in coral genera that are 𝛼-proteobacteria dominated, and once in coral genera that are γ-proteobacteria dominated. Both datasets were visualized with ancestral state reconstruction (Figs. S2A, B). Correlations between microbiome dominance and disease were visually apparent only in reconstructions of the γ-proteobacteria dominated corals, and the dominance-disease correlation was far stronger in γ-proteobacteria dominated corals (PGLS: R^2^ = 0.50, p = 0.0001, FDR q =0.003; Supplementary Data Table 3c), where dominance explained most (50%) of the variation in disease susceptibility. In contrast, 𝛼-proteobacteria dominated tissue microbiomes showed no discernable dominance-disease correlations either visually or statistically (PGLS: R^2^ = 0.06, p = 0.31, FDR q = 0.81; Supplementary Data Table 3c). This suggested that overall dominance-disease correlations are unlikely to be driven by 𝛼-proteobacteria, but may be driven by γ-proteobacteria or specific taxa within this bacterial class. Critically, nothing about these results contradicts the possibility that some 𝛼-proteobacteria are coral pathogens, parasites, or opportunists^5^. It merely suggests that in healthy corals, dominance by 𝛼-proteobacteria does not predict the overall level of disease susceptibility of coral genera, whereas dominance by one or more γ-proteobacteria does.

**Beta diversity explains little variation in disease susceptibility.** Animal microbiomes are often conceived of as having some compositions that are associated with health, and others that are dysbiotic or unhealthy. We sought to test whether this same microbiome beta-diversity framework could predict the extent to which healthy members of different coral taxa are vulnerable to disease. To do so, we correlated coral disease susceptibility against the top three principal coordinate (PC) axes from Weighted and Unweighted UniFrac analyses of microbiome beta-diversity. In contrast to the strong association between microbiome dominance and disease, microbial community composition had less pronounced associations with disease susceptibility. Weighted UniFrac PC axis 3 only nominally significantly correlated with disease susceptibility in all compartments, but this relationship did not remain significant after accounting for multiple comparisons (PGLS: R^2^ = 0.26, p = 0.04, FDR q = 0.90; Supplementary Data Table 4).

**Coral opportunist abundance in healthy corals does not predict genus-wide disease susceptibility.** Correlations between *Endozoicomonas* and disease across the coral tree were initially surprising, as *Endozoicomonas* is not thought to be associated with coral pathogenesis. This raised the question of whether the abundance of known or suspected coral pathogens in apparently healthy corals correlates with cross-genus differences in disease susceptibility.

The abundance of bacterial groups containing prominent putative bacterial pathogens (such as *Vibrionales, Nostocales* or *Rickettsiales*, see Vega Thurber and co-authors^6^) in healthy corals did not show any correlation with disease susceptibility among coral species when tested (Supplementary Data Table 6). Thus, having high abundances of coral opportunists when healthy does not seem to be a hallmark of disease-susceptible corals. This is mostly expected since the abundance of pathogens typically only increases during stress. These observations in healthy corals leave open the question of what about *Endozoicomonas* causes it to be so strongly correlated with coral disease susceptibility.

**Testing for other associations between dominant microbes and disease.** After testing *Endozoicomonas -* disease associations as a prior hypothesis, we also sought to put these associations in context by testing for correlations with disease in all other dominant microbial genera found in the study (Fig. 2C; Supplementary Data Table 7a). This scan confirmed that *Endozoicomonas* showed far stronger correlations with disease than other microbes in tissue, mucus, and all compartments together. One additional dominant genus in coral skeleton, *Paramaledivibacter* (Phylum Firmicutes/Bacillota), also correlated with disease susceptibility, though this correlation was only nominally significant (PGLS: R^2^ = 0.13, p = 0.018, FDR q = 0.282; Fig. 2C).

We also reran this analysis with all zero relative abundance counts excluded (Supplementary Data Table 7b) to assess whether changes in the abundance of dominant microbes, once they were already present, showed the same or different patterns as the overall analysis. The abundance of Endozoicomonas overall (PGLS: R^2^ = 0.28, p = 0.0005, FDR q = 0.01), in mucus (R^2^ = 0.42, p = 0.00008, FDR q = 0.004), or in tissue (R^2^ = 0.35, p = 0.0001, FDR q = 0.004) again showed significant associations with disease, while its abundance in skeleton did not (R^2^ = 0.04, p = 0.23, FDR q = 0.95). No other lineages showed significant associations with disease after correction for multiple comparisons. Thus, even considering only coral genera that host *Endozoicomonas,* the relative abundance of this microbe correlates with disease susceptibility.

**Other microbes associated with growth rate.** We sought to contextualize our results on *Endozoicomonas* and growth rate by examining whether other dominant microbes had similar associations with changes in coral growth rate over evolution (Supplementary Data Table 9a, b, Fig. S3). This analysis revealed several other microbes whose relative abundance in corals where they were present (i.e., excluding zero counts) correlated with coral growth rate. Uncultured Rhodospirillales in family Terasakiellaceae (phylum: Proteobacteria) were strongly and positively correlated with growth in all compartments. Conversely, the relative abundance of *Candidatus* Nitrosopumilis (phylum: Thaumarchaeota) overall or in coral tissue was negatively correlated with growth rate. Finally, the relative abundance of *Enhydrobacter* (phylum: Proteobacteria) in coral mucus was positively correlated with growth.

As in our prior *Endozoicomonas-*specific analysis, we repeated these tests including zero counts in order to account for both initial establishment of symbiosis and later increases or reductions in abundance (rather than changes in abundance only). The results were similar, except that *Pseudomonas* in mucus and uncultured Myxococcales in skeleton were significantly associated with growth rate, while *Endozoicomonas* and *Enhydrobacter* were not. Thus, associations between host traits and the microbiome may in some cases depend only on presence or absence, while in other cases like *Endozoicomonas* these host-microbiome associations may track expansions or contractions in microbial relative abundance.

Overall, these results suggest that while several microbial taxa correlate positively or negatively with coral growth rates, *Endozoicomonas* appears unique in its association with both growth and disease.

**References**

1. Stachowicz, J. J., Whitlatch, R. B. & Osman, R. W. Species diversity and invasion resistance in a marine ecosystem. *Science* **286**, 1577–1579 (1999).

2. Morais, J., Cardoso, A. P. L. R. & Santos, B. A. A global synthesis of the current knowledge on the taxonomic and geographic distribution of major coral diseases. *Environmental Advances* **8**, 100231 (2022).

3. Klinges, G., Maher, R. L., Vega Thurber, R. L. & Muller, E. M. Parasitic ‘*Candidatus* Aquarickettsia rohweri’ is a marker of disease susceptibility in *Acropora cervicornis* but is lost during thermal stress. *Environmental Microbiology* **22**, 5341–5355 (2020).

4. Baker, L. J. *et al.* The coral symbiont *Candidatus* Aquarickettsia is variably abundant in threatened Caribbean acroporids and transmitted horizontally. *ISME J* **16**, 400–411 (2022).

5. Pantos, O. *et al.* The bacterial ecology of a plague-like disease affecting the Caribbean coral *Montastrea annularis*. *Environmental Microbiology* **5**, 370–382 (2003).

6. Vega Thurber, R. *et al.* Deciphering Coral Disease Dynamics: Integrating Host, Microbiome, and the Changing Environment. *Frontiers in Ecology and Evolution* **8**, (2020).

**
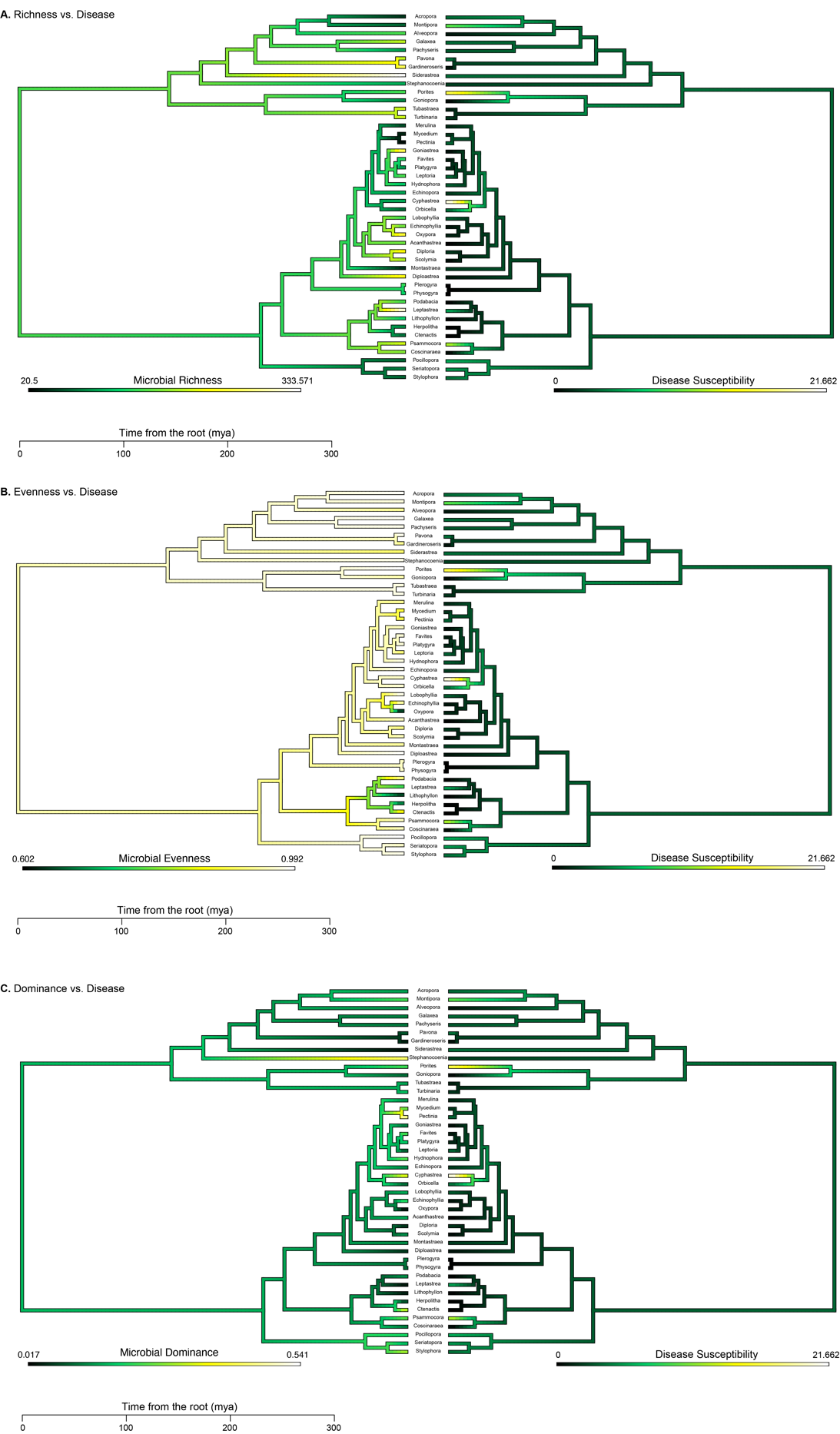
**

**Fig. S1.** Ancestral State Reconstruction of microbiome diversity and disease**.** Ancestral state reconstructions show disease susceptibility (right) and microbial alpha diversity metrics (left), including A) species richness, B) evenness (Gini Index), and C) dominance (Simpson’s Index).


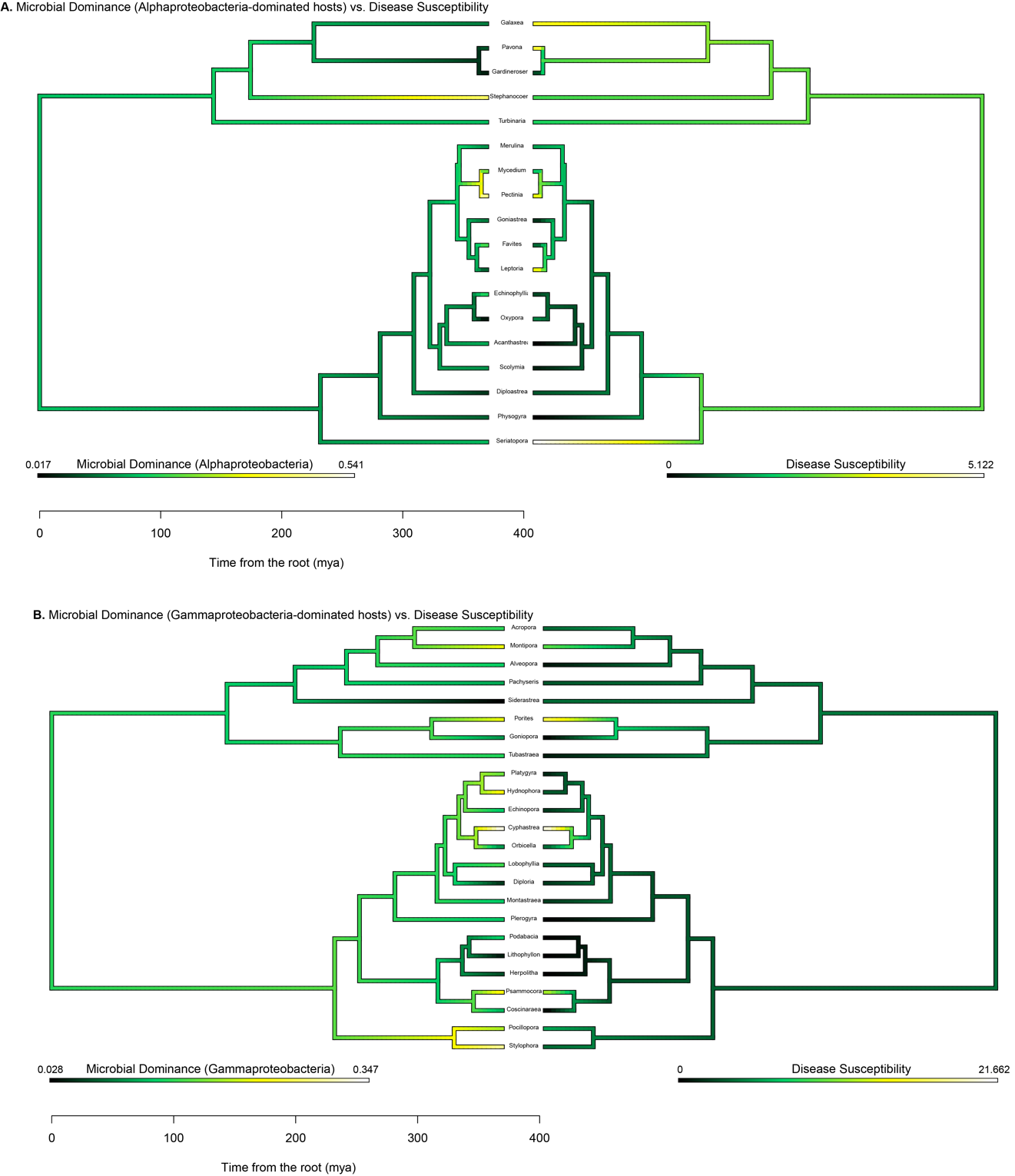


Fig. S2. Ancestral State Reconstruction of microbiome diversity and disease by dominant microbial class. Ancestral state reconstructions showing disease susceptibility (left) and microbial alpha diversity metrics (right), including A) species richness, B) evenness (Gini Index), and C) dominance (Simpson’s Index).


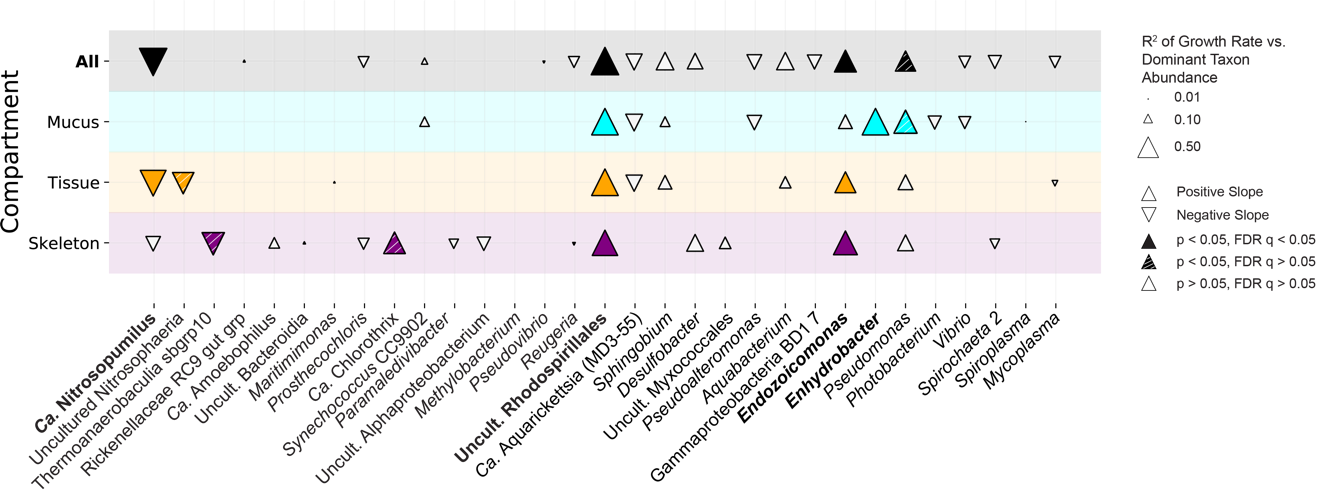
Fig. S3. Coral growth rate vs. dominant taxon relative abundance (zeros excluded). Arrows indicate positive (up) or negative (down) correlations between microbial relative abundnace and growth rate. In this analysis, only corals where a microbe was observed in at least 1 count were included in correlations in order to isolate the effects of expansions or contractions of microbial relative abundance where symbiosis has already been established. Arrow size reflects the R^2^ of the correlations between average coral host growth rate and dominant taxon relative abundance in corals. Filled arrows refer to significant correlations, striped arrows indicate nominally significant correlations (did not pass False Discovery Rate control for multiple comparisons) and open arrows indicate correlations that are not significant. See Supplementary Data Table 9b for details.


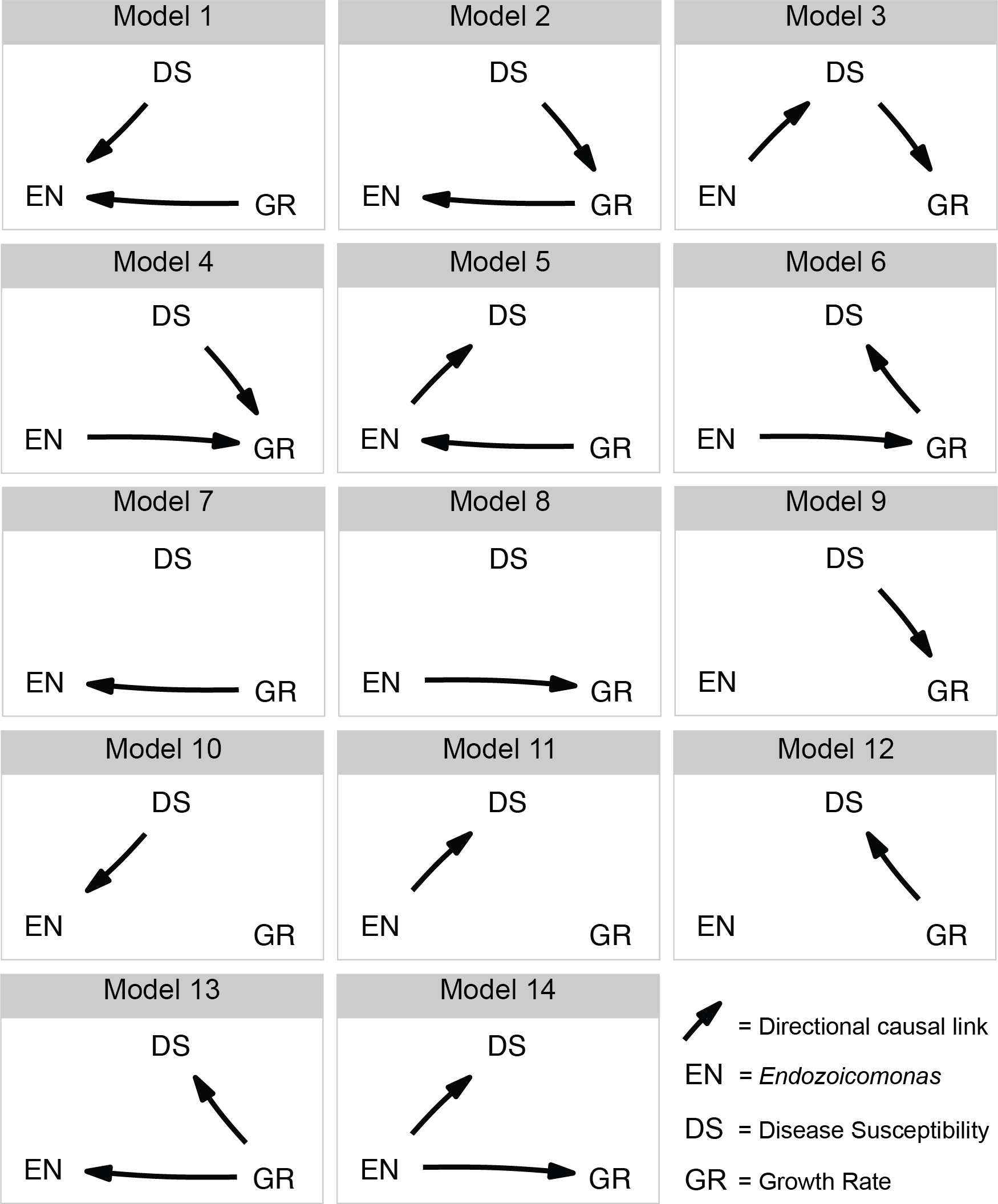


Fig. S4. Model selection for phylogenetic causality analysis. Arrows indicate the direction of causal links, with an arrow from A to B indicating A causes B. These models represent the fourteen plausible causal pathways that were tested in our phylogenetic causality analysis. EN = *Endozoicomonas* relative abundance, DS = coral disease susceptibility, and GR = coral growth rate.


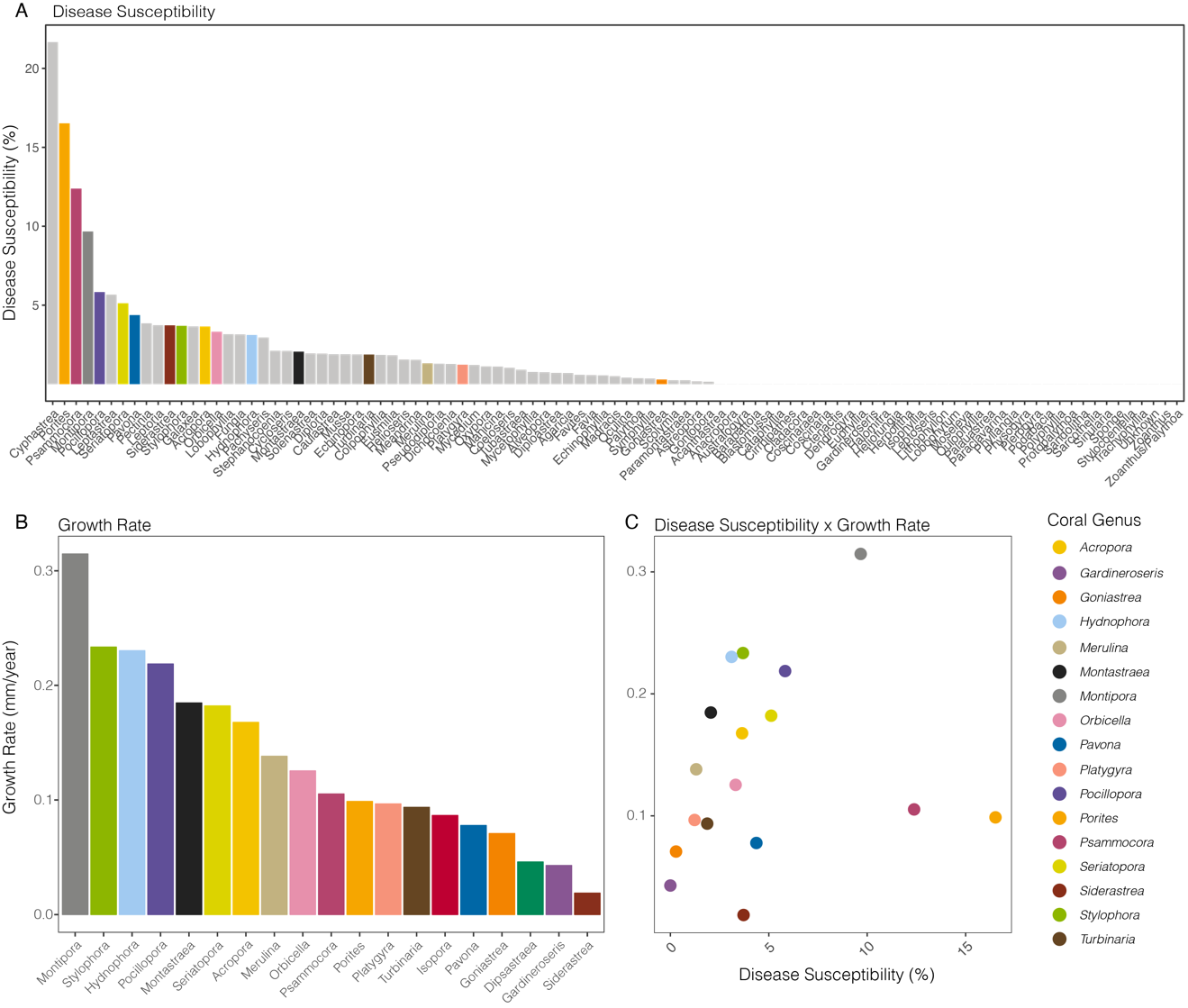


Fig. S5. A) Coral host disease susceptibility, B) growth rate, and C) disease susceptibility plotted against growth rate, by coral host taxon (genus level). Colored bars and points in all three panels correspond to taxa with available growth rate data.
